# Simple Cholecystectomy Is Adequate for Patients With T1b Gallbladder Adenocarcinoma < 1 cm in Diameter

**DOI:** 10.3389/fonc.2019.00409

**Published:** 2019-05-22

**Authors:** Zhengshi Wang, Yao Li, Wenli Jiang, Jie Yan, Jiaqi Dai, Binghua Jiao, Zhiqiang Yin, Yun Zhang

**Affiliations:** ^1^Thyroid Center, Shanghai Tenth People's Hospital, Tongji University School of Medicine, Shanghai, China; ^2^Shanghai Center for Thyroid Diseases, Shanghai, China; ^3^Department of Rehabilitation Medicine, The 960th Hospital of the PLA, Jinan, China; ^4^Department of Biochemistry and Molecular Biology, The Faculty of Basic Medical Science, Second Military Medical University, Shanghai, China

**Keywords:** gallbladder adenocarcinoma, lymph node metastasis, T1b, SEER, simple cholecystectomy, extended cholecystectomy

## Abstract

**Purpose:** Consensus-based clinical guidelines recommend that simple cholecystectomy (SC) is adequate for T1a gallbladder adenocarcinoma (GBA), but extended cholecystectomy (EC), SC plus lymphatic dissection, should be considered for T1b and more advanced GBA. Whether lymphatic dissection is necessary for the treatment of T1b GBA remains controversial. This study attempts to better define the current criteria for local treatment of T1b GBA, by examining the relationship between lymph node (LN) metastasis and tumor size in such patients.

**Patients and methods:** Clinical data from patients with T1b GBA receiving curative surgical treatment between 2004 and 2015 were collected from the Surveillance, Epidemiology, and End Results (SEER) database. Baseline characteristics for the entire cohort were described, and overall survival (OS) and cancer-specific survival (CSS) were analyzed with the Kaplan–Meier method.

**Results:** In total, 277 patients were enrolled for further analysis; 127 underwent lymphadenectomy. Among them, 23 patients had tumors <1 cm in diameter, none of which had LN metastasis; 104 patients had tumors ≥1 cm, 15 of which had positive LNs. In the group with tumor size <1 cm, there was no significant survival difference between treatment with SC or EC (*P* = 0.694). A clinical benefit was observed in T1b GBA patients with a tumor size ≥1 cm receiving EC vs. those receiving SC (*P* = 0.012).

**Conclusion:** SC was adequate for treatment of T1b GBA < 1 cm in diameter. This evidence may be included as part of current guidelines.

## Introduction

Gallbladder cancer is a rare malignancy with an incidence of 1.13/100,000 (1). This fatal disease has a high mortality rate (2), resulting in an overall 5-year survival rate of <5% (3). Gallbladder adenocarcinoma (GBA) is the most common subtype of gallbladder cancer, accounting for ~76–90% (4, 5). The National Comprehensive Cancer Network (NCCN) clinical guidelines recommend simple cholecystectomy (SC) to be adequate for the treatment of T1a (mucosal involvement) GBA since it is a regional disease (6). However, for T1b (muscular involvement) and more advanced GBA, extended cholecystectomy (EC), including lymph node (LN) dissection, should be considered (6–9). Remarkably, some reports have found no clinical benefit to T1b GBA patients receiving EC vs. SC (10–13). The need for LN dissection in T1b GBA patients remains controversial; therefore, a well-defined tumor index that considers LN metastasis in T1b GBA is urgently needed.

As part of the American Joint Committee on Cancer (AJCC) tumor-lymph-metastasis (TNM) staging system, the T category of hollow viscera tumors (e.g., stomach, intestines, and gallbladder) describes vertical tumor penetration; the effect of horizontal tumor extent (tumor size) is not considered. Gotoda et al. (14) reported a strong association between tumor size larger than 3 cm and LN metastasis in early gastric cancer. In colorectal cancer, tumors exceeding 4.5 cm were also found to be associated with high N classification (15). The relationship between tumor size and LN metastasis in gallbladder cancer, to our best knowledge, has not yet been elucidated. This study was performed to evaluate the relationship between tumor size and LN metastasis in T1b GBA to provide more optimal treatment.

## Materials and Methods

### Ethics Statement

This study was approved by the institutional review board of Shanghai Tenth People's Hospital, Tongji University School of Medicine. Patients from the Surveillance, Epidemiology, and End Results (SEER) database had previously consented to participate in any scientific research worldwide.

### Patients

**Figure 1:**
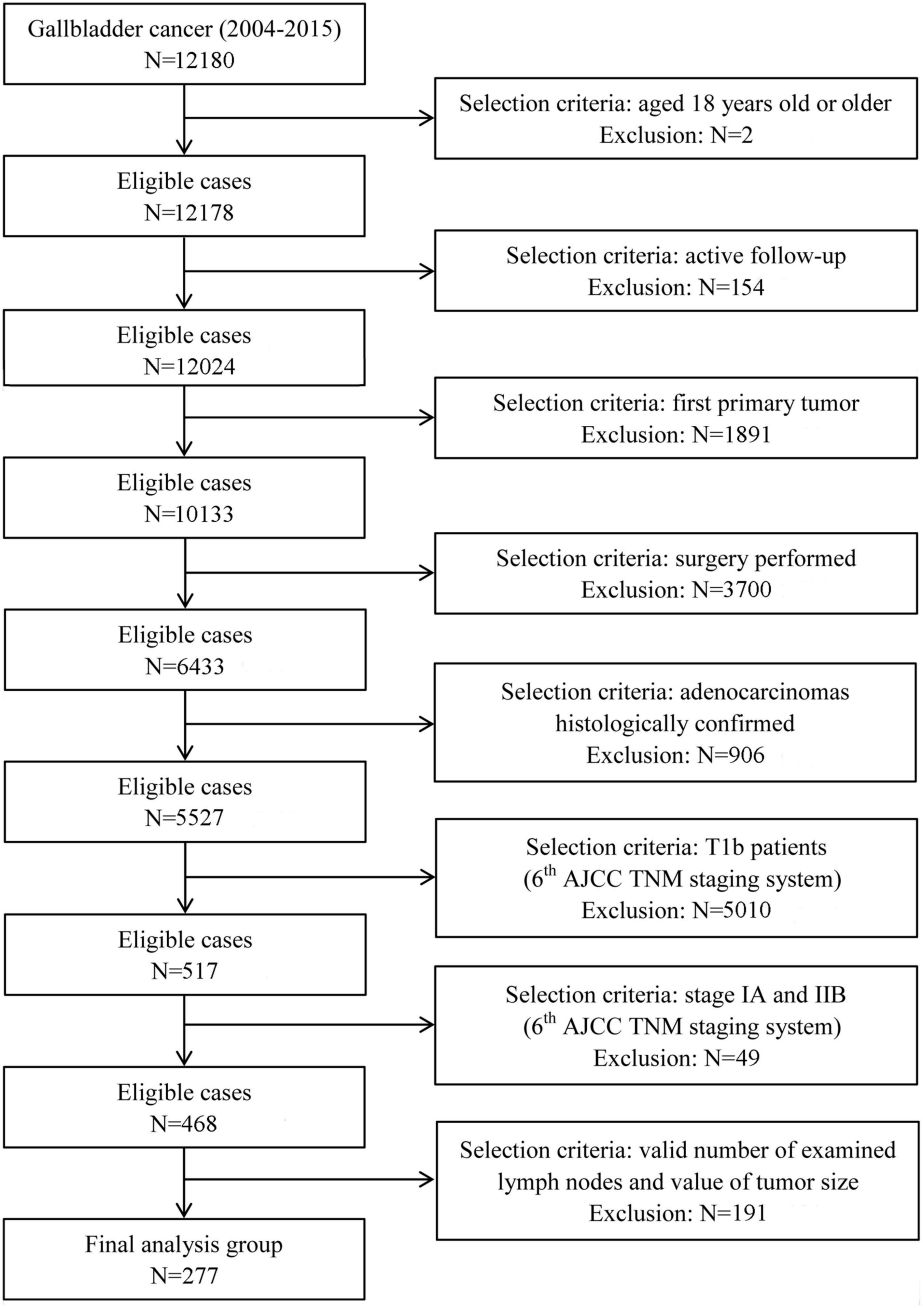
Patient selection flowchart.

T1b GBA was defined as an adenocarcinoma confined to the muscular layer of gallbladder. All patients with T1b GBA were collected from the SEER database, the largest publicly available cancer dataset in the United States (16). Information related to T1b GBA at diagnosis was available for patient data registered between 2004 and 2015. Only patients enrolled after 2004 were collected because depth of tumor invasion was not recorded before 2004 in the SEER database. All T1b patients were uniformly staged according to the 6th or 8th edition of the American Joint Committee on Cancer (AJCC) staging manual because both share the same definition of T1b category (17, 18). Patients with distant metastasis (stage IV) were not eligible because surgical treatment was not the standard therapy. Detailed selection and exclusion criteria are shown in Figure 1.

### Statistical Analysis

Absolute number and incidence of T1b GBA were calculated according to tumor size, age, grading, gender, race/ethnicity, marital status, and status of LNs. The number of patients with positive LNs was the frequency of T1b GBA patients diagnosed with positive LNs. Race/ethnicity representation among our study cohort included those patients identified as White, Black, and Other race/ethnicity. Tumors were staged according to the guidelines outlined in the 8th edition of the AJCC staging manual. Kaplan–Meier survival curves were generated to analyze overall survival (OS) and statistical significance was considered to be two-sided *P* < 0.05.

## Results

### Patient Characteristics

In total, clinical data from 277 patients in the SEER database with pathologically diagnosed T1b GBA were included in this study (Table 1). The median age at diagnosis was 69 years (range, 37–95 years). The proportion of females (71.5%) was higher than males (28.5%). Race-adjusted GBA incidence was 66.4% within the white ethnicity, followed by other (Asian or Pacific Islander, American Indian/Alaska Native, and unknown race/ethnicity) (17.0%) and Black (16.6%).

**Table 1:** Demographic and tumor characteristics of GBA patients from the SEER database (*n* = 277).

**Characteristics**	**No.(%)**
**MEAN TUMOR SIZE (range, mm)**	26.7(1-80)
GRADING^*^	
I+II	220(79.4%)
III+IV	48(17.3%)
**MEDIAN AGE (range, years)**	69(37–95)
**GENDER**	
Male	79(28.5%)
Female	198(71.5%)
**ETHNICITY**	
White	184(66.4%)
Black	46(16.6%)
Other	47(17.0%)
**MARITAL STATUS**	
Married	146(52.7%)
Single/Widowed/Divorced/Separated	115(41.5%)
Other	16(5.8%)
**STATUS OF LNs**	
negative(stage IA^**^)	262(94.6%)
positive(stage IIB^**^)	15(5.4%)
**LYMPHADENECTOMY**	
No	150(54.2%)
Yes	127(45.8%)
Mean No. of evaluated LNs if Yes(range)	3.9(1-80)

* *9 cases missing*.

** *According to the 6th AJCC TNM staging system*.

### Association Between LN Metastasis and Tumor Size

**Figure 2:**
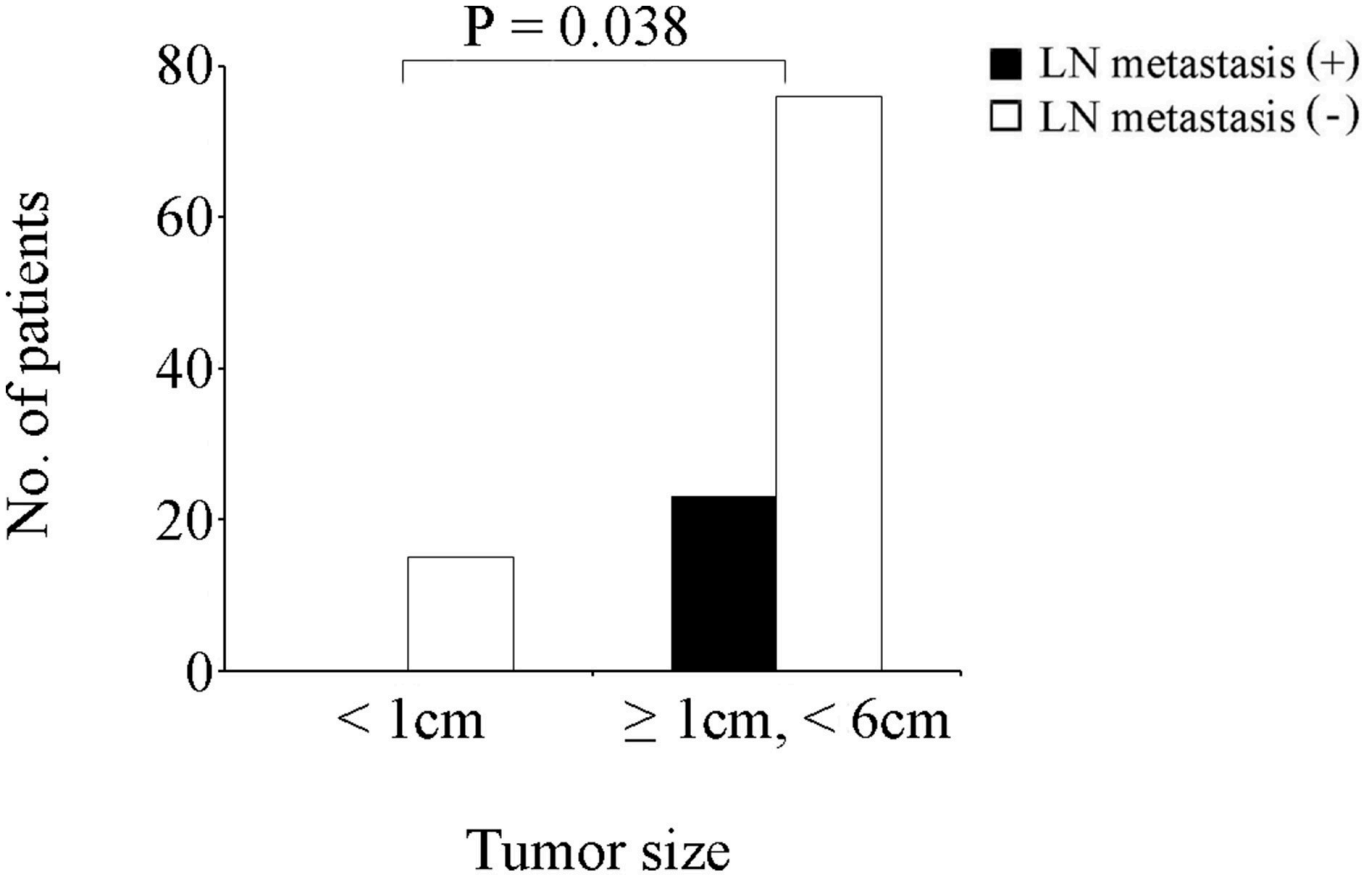
Comparison of GBA patients receiving EC according to LN status and tumor size.

Of the 277 patients with T1b GBA, 127 (45.8%) underwent EC, while 150 (54.2%) patients underwent SC. Additionally, among the 127 patients receiving EC (Table 2), 23 (18.1%) patients had tumors <1.0 cm in diameter, none of which had positive LNs. Three (11.1%) patients had positive LNs in the group of tumors ≥ 1.0 cm and < 2.0 cm in diameter; six (23.1%) had positive LNs in the group of tumors ≥2.0 cm and <3.0 cm in diameter; two (10.5%) had positive LNs in the group of tumors ≥ 3.0 cm and < 4.0 cm in diameter; three (23.1%) had positive LNs in the group of tumors ≥ 4.0 cm and 5.0 cm in diameter; one (16.7%) had positive LNs in the group of tumors ≥5.0 cm and <6.0 cm in diameter; none had positive LNs in group of tumors ≥6.0 cm in diameter probably due to the small sample. Thus, patients with tumors <1.0 cm and those with tumors ≥1.0 cm and <6.0 cm were compared with Fisher's Exact Test and it was found that the latter had a higher rate of LN metastasis (Figure 2, *P* = 0.038).

**Table 2:** Proportion of patients with positive LNs at each tumor size interval.

**Patients with lymphadenectomy**	**SEER (*****n*** **=** **127)**								
Tumor size(mm)	1-9	10-19	20-29	30-39	40-49	50-59	60-69	70-79	80-160
No. of total patients	23	27	26	19	13	6	3	4	6
No. of patients with positive LNs(%)	0	3 (11.1%)	6 (23.1%)	2 (10.5%)	3 (23.1%)	1 (16.7%)	0	0	0
Mean no. of evaluated LNs (Range)	4.2 (1-26)	2.6 (1-7)	3.7 (1-8)	6.7 (1-80)	3.2 (1-12)	3.2 (1-6)	1 (1)	2 (1-5)	5.8 (1-17)

### Survival Analysis

**Figure 3:**
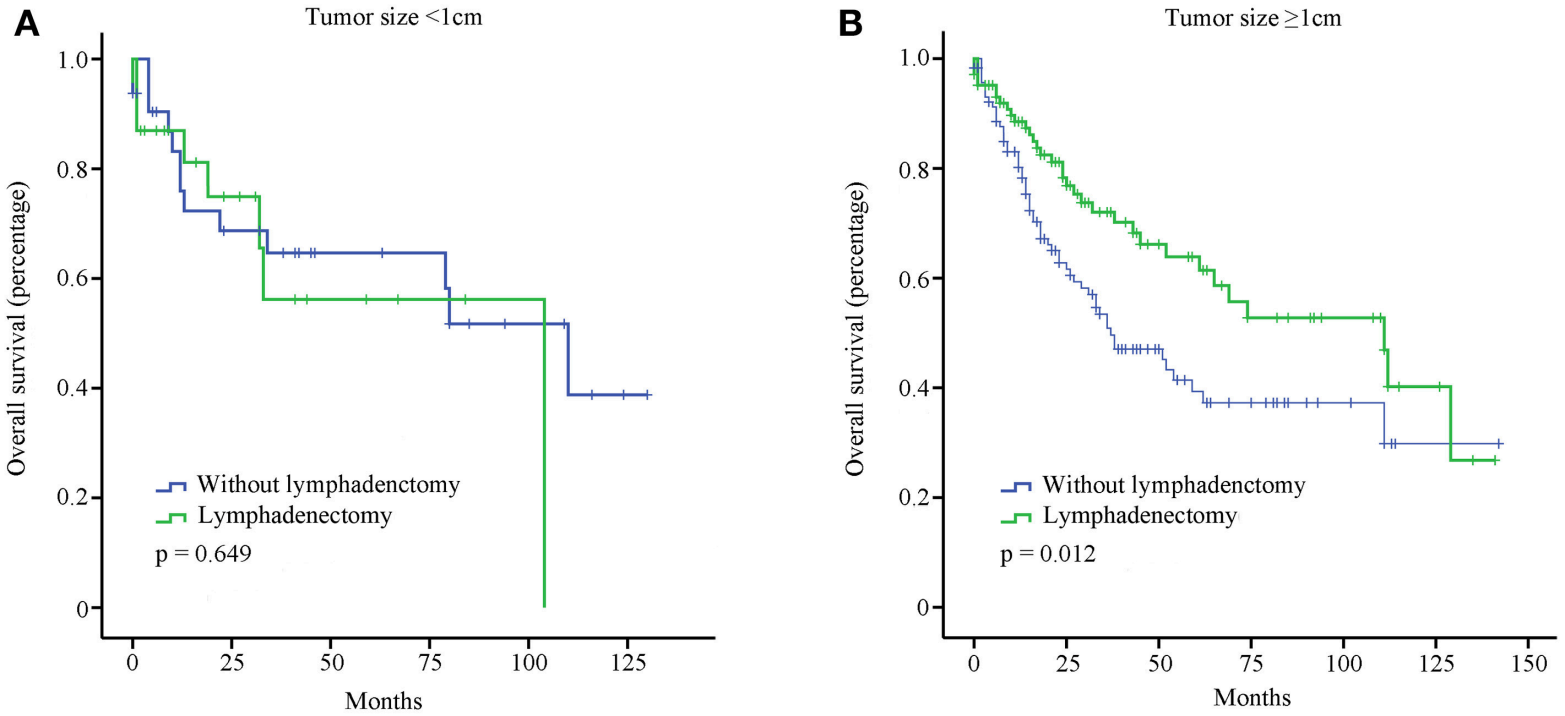
OS of GBA patients stratified by tumor size and surgical treatment using Kaplan-Meier analysis. **(A)** Tumor size <1 cm; **(B)** Tumor size ≥1 cm.

OS of T1b GBA patients receiving SC or EC are presented in Figure 3. Compared with SC treatment, EC did not prolong the OS of T1b GBA patients with tumor size < 1 cm (*P* = 0.694). Clinical benefit was observed in T1b GBA patients with tumor size ≥1 cm receiving EC vs. those receiving SC (*P* = 0.012).

## Discussion

The present study evaluated the number of positive LNs in patients with T1b GBA and the relationship between tumor size and LN metastasis. There were no positive LNs observed in T1b GBA with tumors <1 cm in diameter. These results indicate that SC is adequate for the treatment of T1b GBA in patients with a tumor <1 cm in diameter. Treatment distinction based on tumor size will minimize thdiameter. These results indicate that SC is adequate for the treatment of T1b GBA in patientse need for reoperation from complications in T1b GBA patients and further extend criteria for local treatment.

LN metastasis is the most common metastatic modality in gallbladder cancer. The rate of such metastasis can be as high as 60–80% in stage T3 or T4 gallbladder cancer (19). LN metastasis is notably infrequent in early gallbladder cancer, however. Kohei Shibata et al. published a retrospective study of 72 patients who underwent macroscopically curative surgical resection for stage T1b–T3 gallbladder cancer, and none of the patients with T1b disease had lymphatic invasion (20). Further, Shirai et al. reported that LN metastasis was rarely found in T1b gallbladder cancer (21). In our population-based study, 5.4% (15/277) of patients with T1b GBA had LN metastasis, but no LN metastasis was observed in patients with T1b GBA < 1 cm in diameter. Similarly, for T1b GBA patients with tumor <1 cm in diameter, significant survival differences were not observed between patients receiving SC and those receiving EC. These findings indicated that patients with T1b GBA < 1 cm in diameter were a population with specific indolent tumor behavior.

Most studies support the NCCN guideline recommendation that EC be performed for T1b GBA (6–9), while other studies have argued SC to be adequate (10–13). We speculate that the controversy may be elucidated by the proportion of T1b GBA <1 cm in diameter to entire T1b GBA cohort. When the proportion is low, there is a significant difference observed between T1b GBA patients receiving SC and those receiving EC. Conversely, when the proportion is high enough, there is no significant difference observed. However, for the treatment of T1b GBA ≥1 cm in diameter, EC is still recommended to improve clinical outcomes.

Although we used the one of the largest cancer patient databases in an attempt to evaluate the relationship between LN metastasis and tumor size, the present findings were limited by its retrospective nature. The sample size of this study is relatively small and more large-scale studies with prospective data are needed to validate these conclusions.

## Ethics Statement

This study was approved by the institutional review board of Shanghai Tenth People's Hospital, Tongji University School of Medicine. Patients from the Surveillance, Epidemiology, and End Results (SEER) database have consented to participate in any scientific researches worldwide.

## Author Contributions

ZW and ZY made substantial contributions to the design of the study, carried out the analysis, interpreted the data. YL, JY and JD contributed to the review of previous literature. WJ and BJ contributed substantially to the data discussion and critically commented on the manuscript for scientific content. YZ and ZW made substantial contributions to the conception and design of the study, data interpretation and drafting of the manuscript, were responsible for the quality of the overall manuscript. All authors approved the final version of the manuscript.

### Conflict of Interest Statement

The authors declare that the research was conducted in the absence of any commercial or financial relationships that could be construed as a potential conflict of interest.

## References

[1] Henley SJ Weir HK Jim MA Watson M Richardson LC. Gallbladder Cancer Incidence and Mortality, United States 1999-2011. Cancer Epidemiol Biomarkers Prev. (2015) 24:1319–26. 10.1158/1055-9965.EPI-15-0199 26070529

[2] Hueman MT Vollmer CM Jr Pawlik TM. Evolving treatment strategies for gallbladder cancer. Ann Surg Oncol. (2009) 16:2101–15. 10.1245/s10434-009-0538-x 19495882

[3] Goetze TO. Gallbladder carcinoma: prognostic factors and therapeutic options. World J Gastroenterol. (2015) 21:12211–7. 10.3748/wjg.v21.i43.12211 26604631 PMC4649107

[4] Henson DE Albores-Saavedra J Corle D. Carcinoma of the gallbladder. Histologic types, stage of disease, grade, and survival rates. Cancer. (1992) 70:1493–7. 10.1002/1097-0142(19920915)70:6<1493::AID-CNCR2820700608>3.0.CO;2-U 1516000

[5] Samuel S Mukherjee S Ammannagari N Pokuri VK Kuvshinoff B Groman A. Clinicopathological characteristics and outcomes of rare histologic subtypes of gallbladder cancer over two decades: a population-based study. PLoS ONE. (2018) 13:e0198809. 10.1371/journal.pone.0198809 29889907 PMC5995371

[6] Benson AB III Abrams TA Ben-Josef E Bloomston PM Botha JF Clary BM. NCCN clinical practice guidelines in oncology: hepatobiliary cancers. J Natl Compr Canc Netw. (2009) 7:350–91. 19406039 10.6004/jnccn.2009.0027 PMC4461147

[7] Søreide K Guest RV Harrison EM Kendall TJ Garden OJ Wigmore SJ. Systematic review of management of incidental gallbladder cancer after cholecystectomy. Br J Surg. (2019) 106:32–45. 10.1002/bjs.11035 30582640

[8] Shirobe T Maruyama S. Laparoscopic radical cholecystectomy with lymph node dissection for gallbladder carcinoma. Surg Endosc. (2015) 29:2244–50. 10.1007/s00464-014-3932-9 25303926

[9] Yoon JH Lee YJ Kim SC Lee JH Song KB Hwang JW. What is the better choice for T1b gallbladder cancer: simple versus extended cholecystectomy. World J Surg. (2014) 38:3222–7. 10.1007/s00268-014-2713-x 25135174

[10] Lee SE Jang JY Lim CS Kang MJ Kim SW. Systematic review on the surgical treatment for T1 gallbladder cancer. World J Gastroenterol. (2011) 17:174–80. 10.3748/wjg.v17.i2.174 21245989 PMC3020370

[11] Kim EK Lee SK Kim WW. Does laparoscopic surgery have a role in the treatment of gallbladder cancer? J Hepatobiliary Pancreat Surg. (2002) 9: 559–63. 10.1007/s005340200074 12541040

[12] Steffen T Ebinger SM Tarantino I Widmann B. Prognostic impact of lymph node excision in T1 and T2 gallbladder cancer: a population-based and propensity score-matched SEER analysis. J Gastrointest Surg. (2019). 10.1007/s11605-019-04175-3. [Epub ahead of print]. 30887297

[13] Kim HS Park JW Kim H Han Y Kwon W Kim SW. Optimal surgical treatment in patients with T1b gallbladder cancer: An international multicenter study. J Hepatobiliary Pancreat Sci. (2018) 25:533–43. 10.1002/jhbp.593 30562839

[14] Gotoda T Yanagisawa A Sasako M Ono H Nakanishi Y Shimoda T. Incidence of lymph node metastasis from early gastric cancer: estimation with a large number of cases at two large centers. Gastric Cancer. (2000) 3:219–25. 10.1007/PL00011720 11984739

[15] Kornprat P Pollheimer MJ Lindtner RA Schlemmer A Rehak P Langner C. Value of tumor size as a prognostic variable in colorectal cancer: a critical reappraisal. Am J Clin Oncol. (2011) 34:43–9. 10.1097/COC.0b013e3181cae8dd 20101166

[16] Herzog CE. Overview of sarcomas in the adolescent and young adult population. J Pediatr Hematol Oncol. (2005) 27:215–8. 10.1097/01.mph.0000161762.53175.e4 15838394

[17] Bertani S Pineau P Loli S Moura J Zimic M Deharo E American Joint Committee on Cancer, American Cancer Society. AJCC Cancer Staging Handbook: from the AJCC Cancer Staging Manual. 6th ed New York, NY: Springer (2002).

[18] Amin MB Greene FL Edge SB Compton CC Gershenwald JE Brookland RK. The eighth edition ajcc cancer staging manual: continuing to build a bridge from a population-based to a more “personalized” approach to cancer staging. CA Cancer J Clin. (2017) 67:93–9. 10.3322/caac.21388 28094848

[19] Kondo S Takada T Miyazaki M Miyakawa S Tsukada K Nagino M. Guidelines for the management of biliary tract and ampullary carcinomas: surgical treatment. J Hepatobiliary Pancreat Surg. (2008) 15:41–54. 10.1007/s00534-007-1279-5 18274843 PMC2794356

[20] Shibata K Uchida H Iwaki K Kai S Ohta M Kitano S. Lymphatic invasion: an important prognostic factor for stages T1b-T3 gallbladder cancer and an indication for additional radical resection of incidental gallbladder cancer. World J Surg. (2009) 33:1035–41. 10.1007/s00268-009-9950-4 19225832

[21] Shirai Y Yoshida K Tsukada K. Early carcinoma of the gallbladder. Eur J Surg. (1992) 158:545–8. 1360827

